# Improving IVF Utilization with Patient-Centric Artificial Intelligence-Machine Learning (AI/ML): A Retrospective Multicenter Experience

**DOI:** 10.3390/jcm13123560

**Published:** 2024-06-18

**Authors:** Mylene W. M. Yao, Elizabeth T. Nguyen, Matthew G. Retzloff, Laura April Gago, Susannah Copland, John E. Nichols, John F. Payne, Michael Opsahl, Ken Cadesky, Jim Meriano, Barry W. Donesky, Joseph Bird, Mary Peavey, Ronald Beesley, Gregory Neal, Joseph S. Bird, Trevor Swanson, Xiaocong Chen, David K. Walmer

**Affiliations:** 1 Department of R&D, Univfy Inc., 117 Main Street, #139, Los Altos, CA 94022, USA; 2 Fertility Center of San Antonio, San Antonio, TX 78229, USA; 3 Gago Center for Fertility, Brighton, MI 48114, USA; 4 Atlantic Reproductive Medicine, Raleigh, NC 27617, USA; 5 Piedmont Reproductive Endocrinology Group, Greenville, SC 29615, USAjpayne@pregonline.com (J.F.P.); 6 Poma Fertility, Kirkland, WA 98034, USA; 7 TRIO Fertility Partners, Toronto, ON M5G 2K4, Canada; 8 My Fertility Center, Chattanooga, TN 37421, USA

**Keywords:** IVF, fertility, IVF conversion, IVF utilization, IVF access, fertility access, IVF outcomes, live birth outcomes, artificial intelligence (AI), machine learning (ML), AI/ML, prognostics tool, prediction model

## Abstract

**Objectives:** In vitro fertilization (IVF) has the potential to give babies to millions more people globally, yet it continues to be underutilized. We established a globally applicable and locally adaptable IVF prognostics report and framework to support patient–provider counseling and enable validated, data-driven treatment decisions. This study investigates the IVF utilization rates associated with the usage of machine learning, center-specific (MLCS) prognostic reports (the Univfy^®^ report) in provider-patient pre-treatment and IVF counseling. **Methods:** We used a retrospective cohort comprising 24,238 patients with new patient visits (NPV) from 2016 to 2022 across seven fertility centers in 17 locations in seven US states and Ontario, Canada. We tested the association of Univfy report usage and first intra-uterine insemination (IUI) and/or first IVF usage (a.k.a. conversion) within 180 days, 360 days, and “Ever” of NPV as primary outcomes. **Results:** Univfy report usage was associated with higher direct IVF conversion (without prior IUI), with odds ratios (OR) 3.13 (95% CI 2.83, 3.46), 2.89 (95% CI 2.63, 3.17), and 2.04 (95% CI 1.90, 2.20) and total IVF conversion (with or without prior IUI), OR 3.41 (95% CI 3.09, 3.75), 3.81 (95% CI 3.49, 4.16), and 2.78 (95% CI 2.59, 2.98) in 180-day, 360-day, and Ever analyses, respectively; *p* < 0.05. Among patients with Univfy report usage, after accounting for center as a factor, older age was a small yet independent predictor of IVF conversion. **Conclusions:** Usage of a patient-centric, MLCS-based prognostics report was associated with increased IVF conversion among new fertility patients. Further research to study factors influencing treatment decision making and real-world optimization of patient-centric workflows utilizing the MLCS reports is warranted.

## 1. Introduction

Safe and efficacious, assisted reproductive technology (ART) has the potential to enable millions more people globally to have a family, yet it continues to be underutilized [1,2,3,4,5,6,7,8,9,10]. For patients contemplating the use of in vitro fertilization (IVF) with their own eggs, IVF with donor eggs, intra-uterine insemination (IUI), or a wait-and-see approach, understanding realistic treatment success probabilities (e.g., excellent and poor) is expected to empower patient decision making [11,12,13]. Indeed, delivering personalized prognostics, an important part of patient-centered care, is challenging for providers in all areas of medicine, even though prognostics counseling is one of the pillars of Western medicine dating back to the Hipppocratic tradition [14,15,16,17,18]. IVF, as used broadly to include the use of ovarian stimulation with or without ICSI, is the most prevalent form of ART and is the focus in the rest of this article.

We established a globally applicable framework for localizing IVF prognostics reports to support patient–provider counseling and enable validated, data-driven treatment decisions. Previously, we reported that validated, AI/ML center-specific (MLCS) models produced more accurate and higher IVF live birth probabilities (LBP) compared to age-based prognosis [19,20,21,22,23]. Data requirements, model training, and validation methods for creating MLCS models that compute a patient’s LBP based on her and her partner’s own health data were described [20,21,22,23]. Recently, we further showed that MLCS models created using individual centers’ data outperformed national registry-based models in their ability to accurately identify more patients with higher IVF live birth probabilities [24]. Further, using AI/ML product design-to-deployment best practices and in collaboration with fertility providers treating patients from diverse demographics and payer mix (including self-pay, government, and private health plans), we developed widely applicable data-model pipelines, methods, and locally adaptable clinical workflow and implementation [19,20,21,22,23,24,25].

Other researchers have reported IVF pre-treatment models designed specifically for counseling patients prior to starting their first IVF cycle or after one or more failed cycles. For example, online calculators have been developed using the US Society for Reproductive Technology (SART) or UK Human Fertilization and Embryology Authority (HFEA) national database without center-specific validation [26,27,28]. Alternatively, other researchers—Qiu et al., 2019; Liu et al., 2013; Cai et al., 2024—have also reported using machine learning to train and validate IVF pre-treatment models with excellent, center-specific validation results [29,30,31]. Finally, some IVF prediction models such as that reported by Wen et al., 2022 contributed to our insights but would not practically be usable for patient counseling in the pre-treatment context, since they required data that are not available prior to starting IVF (e.g., number of oocytes, number of blastocysts, etc.) [32]. A discussion of the similarities and differences of the above-mentioned IVF pre-treatment models necessarily relate to model design, training techniques, validation, and performance metrics, which are beyond the scope of this article but are provided in a review by Yao et al., *submitted* [33]. Aside from AI/ML applications in patient counseling, there has been significant development in AI applications using unstructured data such as images or videos to support embryologists in selecting embryos most likely to result in successful pregnancies, and early prototypes have been reported to support optimization of various aspects of the IVF treatment protocol [34,35].

Though IVF success prediction model development has been reported by others, we are not aware of scientific reports of real-world clinical usage of provider–patient prognostic counseling supported by such models and their impact on patients’ treatment decisions [26,27,28]. However, unlike therapeutic intervention, which is evaluated in a progressive, structured set of clinical trials, the development of an AI/ML platform delivering provider- and patient-facing prognostics information must satisfy real-world utilization requirements varying across cultures, socioeconomic environments, and geographies, in addition to scientific validation, before it can be subject to prospective evaluation. Challenges notwithstanding, the reporting of real-world, early-stage clinical usage of AI tools in the medical research literature is important in engaging clinicians to partake in and contribute to technology evolution in medicine. The DECIDE-AI guidelines ensure appropriate and responsible reporting while recognizing requirements—such as customization of implementation in different locations or addressing the diverse needs of clinical teams working at different organizations—unique to technology implementation [36]. For reference, other guidelines adapted for AI-related studies (e.g., TRIPOD-AI, STARD-AI, PRISM-AI, CONSORT-AI, and SPIRIT-AI) address the scientific aspects of prediction modeling or clinical trials unaffected by customized, multisite implementation variations [37,38,39,40,41]. Also, the DECIDE-AI guidelines can apply to diagnostic, prognostic, or therapeutic AI tools and do not impose a particular study design [36].

### Current Research Objectives

The overarching goal of this study was to share real-world, early-stage clinical usage of a patient counseling report conveying personalized IVF LBP validated by ML-trained prediction models for each participating center (i.e., the Univfy^®^ PreIVF Report, a.k.a. Univfy report). Specifically, we aimed to report the relationship between Univfy report usage and first IVF and/or IUI usage (a.k.a. IVF or IUI conversion) at three different time intervals after the new patient visit and assess the relationship between Univfy report usage and IVF and/or IUI conversion. Using the DECIDE-AI guidelines, we conducted this retrospective cohort study with seven unrelated North American fertility centers operating in 17 locations across seven US states and Ontario, Canada. In aggregate, these centers performed ~3500 IVF cycles (excluding embryo transfers) and evaluated ~12,000 new fertility patients annually during the study period spanning 2016–2022. We also describe a stand-alone method for quantifying treatment conversion rate that can be applied to any center to facilitate reporting and comparisons.

## 2. Materials and Methods

### 2.1. De-Identified Data and Data Sources

De-identified data (including clinical service utilization, treatment, and outcomes data) previously linked and processed as part of Univfy client services were entered into the Univfy research database as per the research protocol, which has an exempt status determined by Institutional Review Board (IRB). The overall time period covered by this study is 2016–2022, within which each center had 4–5 years of post-launch usage.

**Figure 1:**
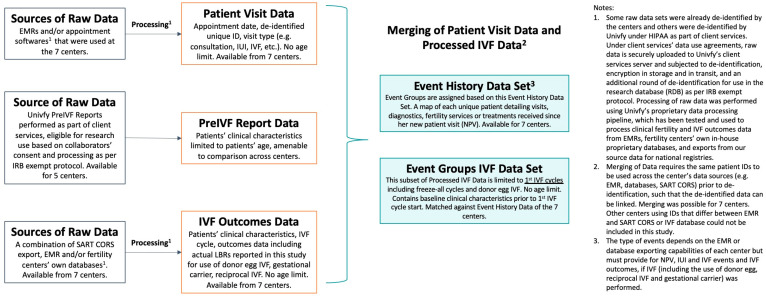
Summary of raw data sources, data processing steps, and the resulting processed and merged data for analysis.

The original data sources provided by each center included any combination of electronic medical record (EMR) systems, appointment data, other third-party software, the Univfy report database, and SART CORS, the US national registry database managed by the Society for Assisted Reproductive Technology (SART) [42]. Figure 1 summarizes the sources of raw data, data processing steps, and the processed and merged data for analysis in addition to additional details. Of the seven centers, one was a Canadian center, and six were American centers that reported to SART.

### 2.2. Univfy Report and MLCS Model Life Cycle

The Univfy report and MLCS model life cycle, including methods used for model training and validation, were reported elsewhere [19,20,21,22,23]. Each center’s own historical outcomes data were initially subjected to model training and validation, as part of HIPAA-compliant, paid services performed independent of and prior to the current research study.

IVF Outcomes Data, used for the development and validation of the prediction model (Figure 1), typically contains age, BMI, at least one ovarian reserve test (e.g., Day 3 FSH, serum anti-mullerian hormone (AMH) levels, antral follicle count (AFC)), clinical diagnoses, male partner history and/or any male factor diagnosis, reproductive history, prior IVF-ET outcomes, and any additional available variables that providers wish to test as model predictors. Of note, AFC was not routinely used or available at the time of IVF pretreatment patient counseling. Other than the patient’s age, no other variables were strictly required for model building, as each center typically has recorded sufficient data for an adequate set of variables to support MLCS model development and validation from consecutive years of data. Subsets of these clinical variables showed relative importance as model predictors, though the predictor subsets and their relative weighting vary slightly across centers [20,23]. Specifically, the important role of AMH and AFC was reported by Nelson et al., 2015 [23].

Related but separate from IVF Outcomes Data requirements is the mandatory versus optional data entry for generating a Univfy report for a patient. Whether a variable is required or optional or not even requested depends on its relative importance in the model and clinical workflow (e.g., whether that clinical variable would realistically be available at the time of patient counseling). For example, prior to 2022, each center was using its own first version of the Univfy report (launched at various times in 2016–2019). Some of those first-version Univfy reports used Day 3 FSH as the only ovarian reserve test or used one or both of Day 3 FSH and AMH. That design reflected (i) IVF Outcomes Data not having sufficient AMH availability from the years prior to the Univfy report launch (i.e., a predictor cannot be used if that predictor was not adequately used in the historical datasets); (ii) AMH not being routinely covered by health insurance plans. Currently, fertility centers are using second-version Univfy reports that all require AMH to be entered, which reflects the adequate AMH usage in the IVF Outcomes Data used to build and validate current models and the current, routine usage of AMH in clinical practice.

The Univfy report provided the personalized probability of having a baby from one, two, or up to three IVF cycles. In addition, the fertility center can elect to also provide the probability of having a baby from an IUI cycle or from one, two, or up to three IUI cycles. The IUI success rates may be cited from a reference article typically used by the providers in that context, or they can be generated from the center’s own IUI data, if available. The Univfy report did not recommend a decision between IUI and IVF. Instead, the report gave prognostic information about both treatment options (if the patient was clinically eligible) to support counseling by the provider and decision making by the patient. Additionally, the Univfy report showed patients how their age, BMI, and ovarian reserve compared with other IVF patients at the same center. Finally, the Univfy report also showed the IVF fees (customized for each center) and, if applicable, showed a cost comparison between pursuing “shared risk” (also called the IVF refund program) and paying fee-for-service.

### 2.3. Defining Event History, Time Interval, and IUI or IVF Treatment Utilization

Using the Event History Data Set, which comprised merged data from Patient Visit Data (including office visits, IUI and IVF treatment dates), Univfy Report Data (including Univfy report usage and associated clinical data), and IVF Outcomes Data (see Figure 1), a chronological Event History was created for each de-identified patient (“patient” hereafter) that had a new patient visit (NPV) during the time period analyzed. The Event History was mapped for 3 timed intervals for analysis: 180 days post-NPV (180D), 360 days post-NPV (360D), and “Ever”. Here, we defined the IVF cycle as having started ovarian stimulation with the intention to perform oocyte retrieval, IVF, or ICSI and subsequent fresh and/or frozen embryo transfer(s). “Conversion” was used to signify the first usage of IUI or IVF following NPV at each center.

IVF conversion included IVF cycles using one’s own or one’s partner’s or third party’s eggs or uterus, egg freezing, and freeze-all and IVF cycles in which oocyte retrieval and/or embryo transfer was cancelled for any reason, outside of illness and other non-fertility related reasons. IUI treatment was taken as-is from the appointment data and included IUI cycles using a partner’s or donor sperm, natural cycle, ovulation induction, ovarian stimulation, ovulation trigger, and/or luteal phase support.

### 2.4. Defining Patient Groups, Univfy Report Usage, Event Groups, and Time Periods for Analyses

**Figure 2:**
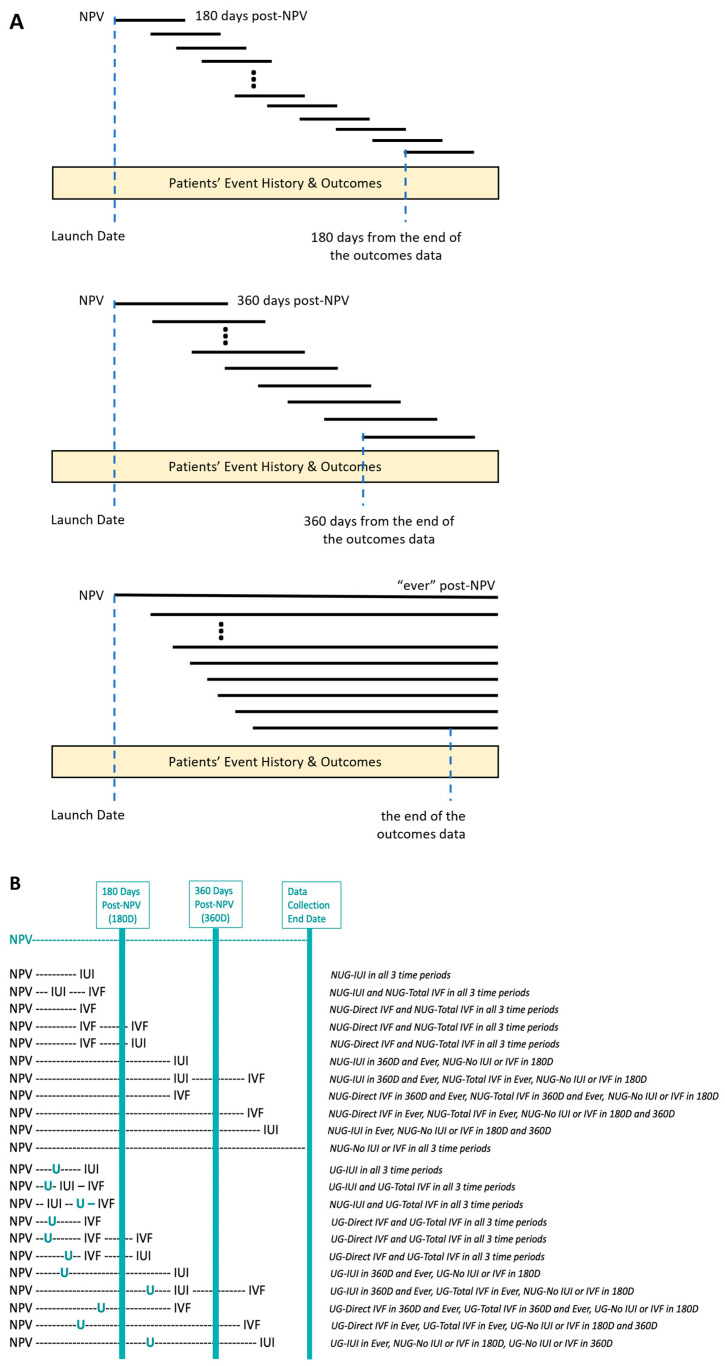
Event History mapping for 3 timed analyses: (**A**) 180 days post-NPV (180D), 360 days post-NPV (360D), and “Ever”. (**B**) Examples showing the mapping of Event History to Patient Groups and Event Groups as per Methods 2.4.

Patients were retrospectively allocated to Patient Groups (Univfy Group vs. No Univfy Group) and Event Groups (IUI, Direct IVF, Total IVF, No IUI, or IVF). The Patient and Event Groups, contemporaneous to each center, and Univfy usage rates were mapped for each of three time intervals: 180D, 360D, and “Ever”. IUI or IVF conversion analyses were performed for each of the three time periods (Figure 2).

**Figure 3:**
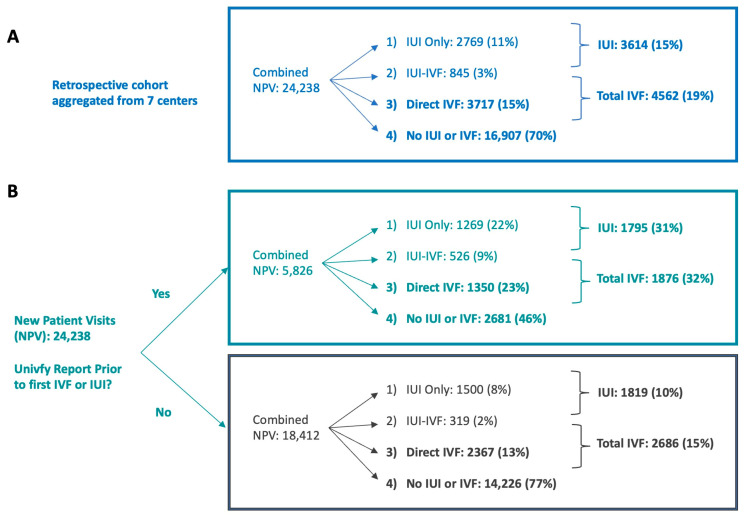
Patient care navigation to treatment conversion by Patient Groups and Event Groups. (**A**) Retrospective patient cohort aggregated from 7 centers and their Event Groups (bolded) based on the Ever timed analysis: new patient visit (NPV)-IUI conversion (IUI Group), NPV-IVF conversion (Direct IVF Group), usage of 1st IVF cycle with or without one or more preceding IUIs (Total IVF Group), and “No IUI or IVF” Group. (**B**) The patient cohort from (**A**) separated into 2 Patient Groups—the Univfy Group (green) and the No Univfy Group (gray).

Patient Groups: The Univfy Group comprised patients who received a Univfy report prior to the first IUI or first IVF usage or had no IUI or IVF subsequent to receiving the Univfy report, within the analysis period (Figure 3A,B). The No Univfy Group comprised patients who did not receive a Univfy report prior to their first IUI or IVF, or at any time during that same analysis period.

Event Groups: Per time period, each patient took one of four mutually exclusive care navigation paths: (1) IUI Only (IUI is her first treatment, and there is no IVF in the same time period); (2) Direct IVF (IVF is her first treatment); (3) IUI-IVF (had IVF after one or more IUIs); (4) “No IUI or IVF”. Event Groups, non-mutually exclusive, were defined by the following navigation paths: (1) IUI conversion (includes IUI Only and IUI-IVF conversion); (2) Direct IVF; (3) Total IVF (includes Direct IVF and IUI-IVF conversion); and (4) “No IUI or IVF” (Figure 3.)

### 2.5. Univfy Report Usage Indications and Contraindications

All 7 centers chose to provide the Univfy report to their patients at no additional cost. Univfy recommended using the Univfy report if the following 2 indications were met and there were no contraindications: (1) the patient must be 18–45 years of age; (2) patient was determined by her provider to be clinically eligible to receive IVF treatment and was interested in being counseled on the benefits and limitations of IVF using their own eggs. Contraindications of the Univfy report implemented during this study period were as follows: (1) patient was in perimenopause or menopause, (2) Day 3 FSH > 15 mIU/mL, or (3) had a prior failed IVF cycle with no blastocysts or euploid blastocysts for transfer, and/or the oocyte or embryo quality was extremely poor. (Univfy offered a separate model addressing patients with past poor embryology outcomes, which is beyond the scope of this article.).

As part of the standard data processing and model development processes, we present the steps and results pertaining to the model and model performance, including indications and contraindications, to the clinical leadership at the center for discussion, any revisions, and final approval. These and any additionally necessary contraindications are then stated explicitly in the Instructions for Use (IFU) manual and then taught to center personnel at the time of training.

### 2.6. Providers’ Autonomy of Univfy Report Usage and Real-World Limitations

Univfy did not impose any additional usage rules. Centers and providers had complete autonomy to choose the extent of Univfy report usage, including the prioritization of Univfy report usage to patients who, in the provider’s clinical judgement, should consider IVF as a first-line treatment for a variety of clinical reasons. During the study period, automated EMR-Univfy data transmission and staffing by advanced practice providers (APP) were not in place at most centers. Therefore, Univfy report usage was limited by staffing and patients’ completion of diagnostic tests.

It was not possible to compare the pre-treatment clinical data between all patients in the Univfy vs. No Univfy Groups. Specifically, baseline pre-treatment clinical data for certain patients would have required de novo EMR data extraction, an activity not permitted under the IRB-exempt study protocol. Patients affected by this data limitation were No Univfy Group patients that took the care navigation paths of IUI Only or “No IUI or IVF”.

### 2.7. Statistical Analyses

Chi-square tests of independence were used to test actual versus expected proportions for Univfy vs. No Univfy report usage and the difference in IUI, Direct IVF, and Total IVF treatment conversions between Univfy and No Univfy Groups at each center. A Wilcoxon signed-rank test was used to test the mean difference in IUI, Direct IVF, and Total IVF treatment conversions across the 7 centers. NS indicated a non-significant *p*-value ≥ 0.05.

Two logistic regression analyses were performed. First, logistic regression was applied to data from all seven centers to test the effect of center and Univfy report usage on the likelihood of each of IUI, Direct IVF, and Total IVF usage. Separately, logistic regression was used to test the effect of center and patients’ age on the likelihood of IVF usage among Univfy Group patients. Per research protocol requirements, the data available for this analysis were limited to Univfy Report Data available between 1 July 2019 and the end of the study period for 5 of 7 centers. We did not extend the regression analysis to test the effect of AMH on IVF conversion, because not all the centers’ first version of the Univfy report (designed and launched at various times in 2016–2019) required AMH usage, as the Univfy report accommodated the use of Day 3 FSH and/or AMH at that time (see Section 2.2).

This study is reported according to the STROBE statement and DECIDE-AI guidelines [36,43].

## 3. Results

### 3.1. Univfy Report Utilization

Univfy report utilization and treatment conversion analysis was performed for all seven centers. The total numbers of unique patients meeting criteria of the three timed analyses were 20,512 (180D), 17,124 (360D), and 24,238 (Ever) in aggregate across the seven centers. The mean Univfy report utilization rates across seven centers for the 180D, 360D, and Ever time periods were 17.4% (95% CI 12.4–22.4%), 20.1% (95% CI 14.5–25.7%), and 24.3% (95% CI 18.3–30.3%), respectively. Since chi-square tests of independence showed that the Univfy report utilization rate was different across centers in each time period assessed (*p* < 0.001), subsequent results were reported for the aggregate data and were either on a per-center level or accounted for “center” as a proxy for center-specific attributes.

### 3.2. Comparison of IUI and IVF Conversion Rates between the Univfy and No Univfy Groups

We tested whether Univfy report usage was associated with differential conversion rates for the Event Groups IUI, Direct IVF, and Total IVF in each timed analysis. Compared to the No Univfy Group, Univfy report exposure increased the odds of IUI conversion by 195% (OR = 2.95, CI 2.63–3.31, *p* < 0.001), Direct IVF conversion by 213% (OR = 3.13, CI 2.83–3.46, *p* < 0.001), and Total IVF conversion by 241% (OR 3.41, CI 3.09–3.75, *p* < 0.001) at 180 days post-new patient visit. Similar findings were observed for the 360D and Ever time periods (Table 1).

**Figure 4:**
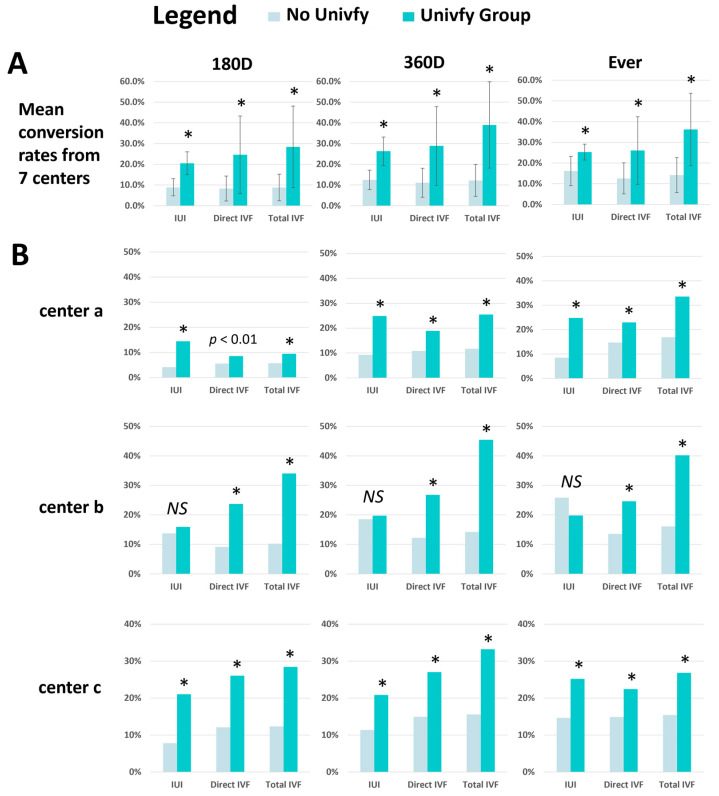

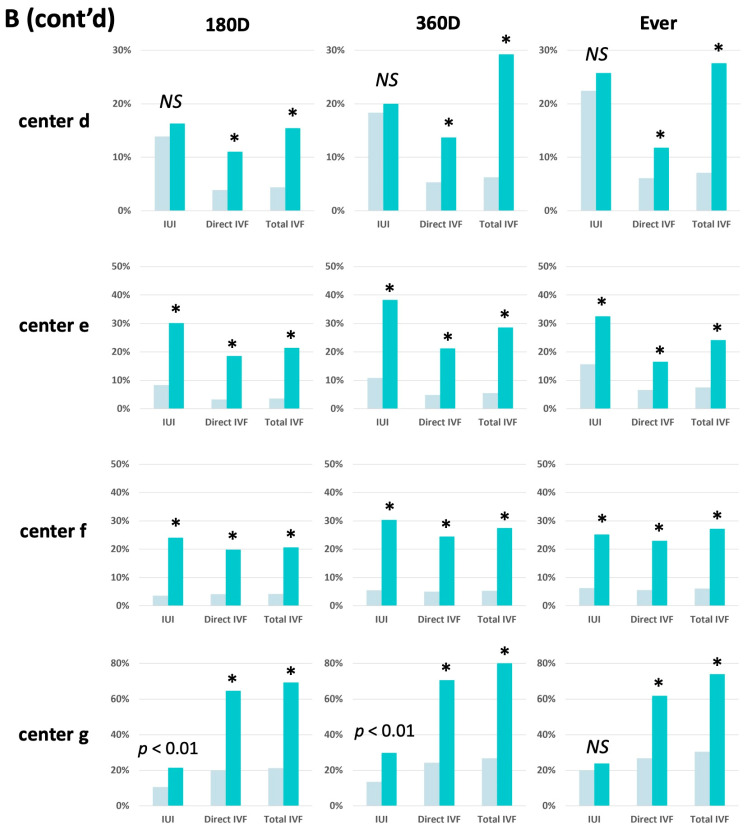
Comparison of IUI, Direct IVF, and Total IVF conversion rates between Univfy Group and No Univfy Group in 3 timed analyses. (**A**) The conversion rates across 7 centers were compared between Univfy Group and No Univfy Group for IUI, Direct IVF, and Total IVF conversions in each timed analyses, 180D, 360D, and Ever, using Wilcoxon signed-rank test. * *p* < 0.05. (**B**) The per-center IUI, Direct IVF, and Total IVF conversion rates are compared using chi-square tests * *p* < 0.001, *NS* not significant.

Wilcoxon signed-rank tests showed that across centers, the Univfy group showed higher conversion rates for IUI, Direct IVF, and Total IVF (Figure 4A). At the per-center level, Direct IVF and Total IVF conversion rates were significantly higher in the Univfy Group in each of the seven centers, and IUI conversion rate was significantly higher in the Univfy Group in five of seven centers (Figure 4B). Using regression models accounting for “center” as a proxy for center-specific attributes, we confirmed that Univfy report usage was a significant predictor for each of IUI, Direct IVF, and Total IVF conversion in each of the time periods assessed (Table 1).

### 3.3. Assessing the Influence of Age on Treatment Decisions within the Univfy Group

Although it was not possible to analyze both Univfy and No Univfy Groups in their entireties with respect to patients’ clinical characteristics, we were able to test within the Univfy Group the potential influence of age on treatment decisions. Specifically, for the Univfy Group, we applied logistic regression to analyze the relationship between center and age on the probability of Direct IVF or Total IVF treatment conversion—using Patient Group and Event Group assignment in the Ever time period. Overall, 2468 patients from five centers in the Univfy Group had data available for this analysis (see restrictions explained in Section 2.7 and Figure 1, Sources of Raw Data for PreIVF Report Data). For context, the Direct IVF and Total IVF conversion rates for those 2468 patients were 31% and 45%, respectively. Using “center” to account for any center-specific attributes, age increased the odds of pursuing Direct IVF (OR = 1.05, CI 1.03–1.07, *p* < 0.001) and Total IVF conversion (OR = 1.03, CI 1.01–1.05, *p* < 0.001). For example, across centers, the average probability of Direct IVF conversion (without doing IUI first) was 30% at age 34 and 37% at age 40, whereas the average probability of Total IVF Conversion (regardless of IUI usage) was 45% at age 34 and 49% at age 40.

## 4. Discussion

Here, we discuss the unique contributions, limitations, and potential biases of this study in the context of addressing patients’ decision support needs using IVF prognostics tools. In addition, we offer insights to improve IVF usage, including ways to evaluate complex care navigation using treatment utilization metrics.

First, we review the intended clinical usage and relevance. The Univfy report was intended to provide pre-treatment IVF success probabilities to support decision making by each patient/couple, with a focus on treatment success expectations. The Univfy report conveyed the personalized IVF success probability based on the IVF cycle start, accounting for cycles that were unsuccessful due to cancellation of egg retrieval, poor ovarian response to stimulation, no embryos to transfer, and pregnancy loss. Though national registries have provided a bird’s eye view of IVF outcomes to inform policy and maternal and newborn safety guidelines, the Univfy report enables providers to communicate the IVF live birth probability based on each patient’s own health data with a prediction model that has been validated for data from their specific fertility center. The practical clinical benefits of using the machine learning, center-specific approach specific to model performance are discussed in other reports and are beyond the scope of this article [19,20,21,22,23,24,25,29,30,31,33].

There were limitations to this study. This study’s retrospective and non-randomized setting likely allowed for a mix of intended and unintended tendencies to use the Univfy report in patients who were more motivated in completing diagnostics and scheduling follow-up visits. Given the constraints of the exempt IRB protocol, it is beyond the scope of this current study to explore full EMR data exports to conduct the most appropriate comparison—Univfy vs. No Univfy Groups—to determine the differences in clinical characteristics of both groups, each in its entirety. Nevertheless, we surmised that even if the size of the “No IUI or IVF” Event Group within the No Univfy Group decreased by 20%, the Univfy Group would still show higher IVF conversion rates. There is also a small risk that not all Univfy reports that were generated were necessarily given to patients or used directly in patient counseling conversations, though providers’ informally self-reported that this issue occurred infrequently.

Potential bias notwithstanding, we learned that of the 2468 patients exposed to the Univfy report prior to their first IUI or IVF (or no treatment), the magnitude of patient’s age as a predictor for IVF conversion was limited after accounting for center as a proxy for all other center-specific attributes. In that regression analysis, we did not analyze AMH effect in addition to age and center effects, because clinical practice changed from using Day 3 FSH to AMH for ovarian reserve evaluation during that time (see AMH usage detailed in Section 2.2). We hypothesize that the use of a the MLCS-based counseling report increases IVF conversion rates by acknowledging personalization, center validation, and transparency in addition to its IVF prognostics delivery. Future studies should test additional factors such as IUI failure and IVF insurance coverage for any independent impact on the IVF cycle start decision. In addition, multicenter analyses and single-center analyses are both important in gaining insights that are generalizable to many centers and recognizing actionable items (a.k.a. low-hanging fruit) specific to each center. These examples highlight the need to shift from AI/ML model building to deployment including efficient and effective use of data to realize the potential of AI/ML to improve medicine and healthcare access and delivery [44]. Whereas most therapeutics can be tested using a “purist” approach using standardized administration (e.g., oral, IV, injections, etc.), studying the clinical impact of AI/ML tools requires additional considerations in the early technology adoption phase. The Univfy report utilization rates showed patterns of cautious adoption of new technology, as typically seen in the broader healthcare field [45].

In hindsight, it may seem limiting to not have full EMR data exports to support the Univfy vs. No Univfy analysis. However, during the study period (2016–2022), this was intentional to focus on establishing model validity, implementation, provider trust, and provider-patient satisfaction. Activities that were not “mission critical” for the model-report usage would have required yet more administrative burden from fertility centers and their clinical teams. The need to prioritize provider effort and overall costs is a necessary discipline in technology development and, in turn, keeps the cost of usage affordable, which directly relates back to making IVF more accessible. Further, there is ongoing adaptation to meet clinical needs. The historical and economic contexts related to AMH usage (Section 2.2) and its impact on model design illustrate the dynamic interactions between clinical practice patterns and AI/ML health technology evolution. The various constraints that we experienced turned out to be quite typical of early-stage studies of AI/ML-based tools implemented to support clinical care. In fact, the DECIDE-AI guidelines were established specifically to provide a framework for appropriate reporting under those constraints [36]. Finally, it would not have been appropriate to initiate a prospective trial back in 2016–2020, before confirming real-world implementation and clinical usage.

At the time of writing, the AI/ML platform offers a white glove service that manages and generates Univfy reports for centers including EMR-integrated automation of MLCS-based prognostics report generation; more fertility centers are also using a wider range of providers, including advanced practice providers (APPs), to scale the accessibility of IVF [35,46]. These types of support, which help to streamline provider team flow and improve patients’ experience, were not in place during the study period. Those and many other support features have assisted providers in adopting routine usage of Univfy reports and are essential for successful technology adoption in the current environment where understaffing is the norm.

Although most fertility centers have internal reporting of IVF utilization rates, there have been no standardized metrics, making inter-center discussions void of “apples-to-apples” relevance. By implementing benchmarks (e.g., Univfy report and conversion metrics such as 180D, 360D, and Ever), fertility centers with very different workflows and demographics could optimize patient-centric care navigation tailored to their teams’ and patients’ needs, while reporting metrics applicable across diverse locations. Also, the Univfy report and benchmark infrastructure further allow us to explore provider–patient shared decision-making in IVF and generate hypotheses for testing. There is an urgent need to understand patient decision making to realize the full potential of fertility care.

## 5. Conclusions

Altogether, this study’s findings suggest that the use of an MLCS-based, IVF prognostics counseling report is associated with higher IVF conversion rates. Among patients having an MLCS-based counseling report, older age was a small yet independent predictor of IVF conversion after accounting for center as a predictor. The retrospective nature of the study did not allow us to test for possible biases that might have caused some patients to receive or not receive the Univfy report. This study’s findings informed the design and implementation of a prospective trial to evaluate the impact of MLCS-based provider–patient counseling on IVF conversion. Further research to study factors influencing treatment decision making and real-world optimization of patient-centric workflows utilizing the MLCS reports is warranted.

## 6. Patents

Univfy Inc. owns or is the licensee of issued and pending patents related to the subject matter of this study, including Patent Number 9,458,495B2, foreign counterparts and patents issued.

## Figures and Tables

**Table 1:** Summary statistics (mean and 95% confidence interval (95% CI)) of the IUI, Direct IVF, and Total IVF conversion rates across 7 centers for Univfy Group and No Univfy Group and the odds ratio of increased conversion in the Univfy Group compared to the No Univfy Group for each timed analysis. Χ^2^ ORs and p-values are based on chi-square test of independence using the aggregate data from 7 centers. Regression OR of Univfy report and *p*-values are based on regression models analyzing the relationship between center and Univfy report exposure on IUI, Direct IVF, and Total IVF conversions.

	Conversion	Mean Conversion Rates across 7 Centers (95% CI)		Χ^2^ Odds Ratio (95% CI, *p*-Value)	Regression OR Univfy Report (95% CI, *p*-Value)
		Univfy Group	No Univfy Group		
180D	IUI	20.5% (16.4–24.6%)	8.9% (5.8–11.9%)	2.95 (2.63–3.31, *p* < 0.001)	3.74 (3.46–4.05, *p* < 0.001)
	Direct IVF	24.6% (10.8–38.5%)	8.3% (3.8–12.7%)	3.13 (2.83–3.46, *p* < 0.001)	2.53 (2.33–2.75, *p* < 0.001)
	Total IVF	28.4% (13.8–43.0%)	8.8% (4.1–13.5%)	3.41 (3.09–3.75, *p* < 0.001)	3.81 (3.37–4.31, *p* < 0.001)
360D	IUI	26.3% (21.1–31.4%)	12.5% (9.0–16.0%)	2.94 (2.63–3.29, *p* < 0.001)	3.61 (3.20–4.06, *p* < 0.001)
	Direct IVF	28.9% (14.9–43.0%)	11.1% (5.9–16.3%)	2.89 (2.63–3.17, *p* < 0.001)	2.67 (2.44–2.92, *p* < 0.001)
	Total IVF	39.0% (23.6–54.4%)	12.2% (6.5–17.9%)	3.81 (3.49–4.16, *p* < 0.001)	4.14 (3.80–4.51, *p* < 0.001)
Ever	IUI	25.3% (22.5–28.1%)	16.2% (10.9–21.5%)	2.05 (1.89–2.23, *p* < 0.001)	2.43 (2.23–2.66, *p* < 0.001)
	Direct IVF	26.1% (14.0–38.2%)	12.6% (7.0–18.2%)	2.04 (1.90–2.20, *p* < 0.001)	2.50 (2.31–2.70, *p* < 0.001)
	Total IVF	36.2% (23.3–49.2%)	14.2% (7.9–20.5%)	2.78 (2.59–2.98, *p* < 0.001)	3.56 (3.31–3.84, *p* < 0.001)

## Data Availability

The datasets used in this study are not readily available due to privacy reasons, as the data were obtained from and owned by different third parties. The corresponding author does not have authorization to share data but will direct your dataset access requests to the appropriate person to obtain permission. The authors welcome requests to support reproducibility testing of methods in other datasets.
